# Isolation and functional identification of three cuticle protein genes during metamorphosis of the beet armyworm, *Spodoptera exigua*

**DOI:** 10.1038/s41598-017-16435-w

**Published:** 2017-11-22

**Authors:** Saad jan, Sisi Liu, Muhammad Hafeez, Xiangmei Zhang, Farman Ullah Dawar, Jiyun Guo, Chao Gao, Mo Wang

**Affiliations:** 1 College of Plant Science and Technology, Huazhong Agricultural University, Wuhan, Hubei 430070 People’s Republic of China; 2 College of Science, Huazhong Agricultural University, Wuhan, Hubei 430070 People’s Republic of China; 3 College of Fisheries, Huazhong Agricultural University, Wuhan, Hubei 430070 People’s Republic of China

## Abstract

The beet armyworm, *Spodoptera exigua* (Hubner), is one of the major crop pests and is a target for current pest control approaches using insecticides. In this study three cuticular protein genes *CPG316*, *CPG860* and *CPG4855* have been cloned from 0 h pupal integument of *S. exigua* through race PCR Strategy. The deduced amino acid sequences were found to contain the RR-2 consensus region of other insect cuticular proteins and construct phylogenetic trees for each protein. Using quantitative RT-PCR, the developmental expression of the three genes through several larval and the early pupal stages was studied. All three genes contribute to the endocuticle although *CPG316* may have a different role from the other two genes. All three newly isolated genes were analyzed and their functions were determined by using direct injection of the dsRNA into early 5^th^ instar larvae. All genes are expressed in the larvae and early pupae but in different patterns. Furthermore, phenotypic results show that these genes have differing effects on the development of cuticle, its flexibility and a big role in metamorphosis in both larval and pupal stages.

## Introduction

The insect cuticle is a multi-layered structure with three functional regions, the epicuticle, the exocuticle, and endocuticle, which differ in protein composition, structure, and functions^1^. The structure of the cuticle is determined by the cuticular proteins. Many types of cuticle show prominent differences in mechanical properties and these differences are related to the properties of the individual proteins^2^. The properties of insect cuticle differ in different stages and species due to the mixture of cuticular proteins. The cuticle performs important roles in many physiological conditions and defends the insect from water loss, penetration of insecticides, and protect from physical injury and pathogens^3–6^. Many conserved motifs in cuticular proteins^5^ have been identified including Rebers and Riddiford (R&R) Consensus^7^. Proteins with R&R Consensus can be split into three groups, RR-1, RR-2, and RR-3, with some correlation to the type or region of the cuticle. The cuticle proteins with 44-amino acid motifs belong to (CPF)^8^, and Tweedle^9^ consensus. Among them, the cuticular protein sequences containing R&R Consensus (CPR) were broadly studied in *Anopheles gambiae, Drosophila melanogaster, Bombyx mori* and *Apis mellifera* by the annotation of genomic data^10–13^. Many cuticular protein genes have been isolated, and their ecdysone-responsive characteristics have been studied^14–20^.

The beet armyworm, *Spodoptera exigua* (Hübner), is a destructive pest of vegetables and crops^14^. This pest has a long history of being treated with insecticides, which have resulted in the development of resistance to a diverse array of chemical classes^21–27^. The life cycle of this pest consists of four different stages: eggs, five larval instars, pupae, and adults^28^. The pupal melanic strain of *Spodoptera exigua* (SEM) with increased fitness was found in a laboratory population^29^. Transcriptome analysis between the SEM strain and wild-type strain (SEW) revealed several cuticular protein genes overexpressed at 0 h pupa stage (unpublished data).

The substitute biotechnological techniques depend mainly on *Bacillus thuringiensis* (BT) expression of insecticidal protein Cry toxins. Nevertheless, there has been a development of resistance to these toxins in some of the species such as (*Heliothis virescens*, Lepidoptera: *Noctuidae*) and (*Ostrinia nubilalis*, Lepidoptera *Pyralidae*)^30,31^. RNAi was first used on the function of insect gene as a highly effective tool for the research purpose of the fruits fly *D. melanogaster* as a model pest^32^. RNAi technology and the elucidation have set for the development and their growth in the RNAi systems and application of this in *D. melanogaster* lately^33^ have reviewed the mechanisms of RNAi uptake in insects body and its possible potential for insect management^34^ RNAi was described by Fire, *et al*.^35^. The post-transcriptional gene silencing ability within target insect with dsRNA double-strand RNAs method was introduced by Geley & Müller^36^.

To advance understanding of the molecular genetics basis of the overexpressed cuticular protein genes in the beet armyworm, we have isolated and characterized three cuticular protein genes from 0 h pupae, as well as the temporal expression profiles of these three genes was observed. The results showed that these three cuticular protein genes had the RR-2 motif consensus sequence based on the conserved motif and phylogenetic tree analysis. The *CPG316* gene was expressed abundantly at the 2 h pupa stage while the *CPG860* and *CPG4855* showed decreasing expression during development from the 3^rd^ instar to the 24 h pupa. The differential expression patterns of these genes may reveal their property to be utilized in a novel control strategy. We used RNAi to observe the function of these genes determined using direct injection of three different concentrations (100 ng/µl, 200 ng/µl and 500 ng/µl) of the dsRNA into early 5^th^ instar larvae. The results revealed that these genes have differing effects on the cuticle in both larval and pupal stages and are required for cuticle development and normal metamorphosis.

## Materials and Methods

### Insects

*S. exigua* larvae from Jingzhou Hubei Province, China, were maintained with artificial diet in the laboratory at 27 °C and 65% relative humidity and a photoperiod of 14L:10D^37^.

### Gene cloning

RNA was extracted from 0 h pupae integument. The cDNA was synthesized as suggested in the SMART RACE kit (Takara, Biotech, Japan). Gene-specific primers (GSP1) and nested gene-specific primers (NGSP2) were designed for 5′- and 3′-RACE using the software Primer Premier 5.0. The primer sequences are listed in (Table S1). The first round PCRs were performed with the GSP primer and Universal Primer Mix (UPM). The 99X diluted first PCR products were used as the templates in the nested PCRs with NGSP. The RACE products were separated on an agarose gel and purified using the AXYGEN Gel and PCR Clean-Up System (Promega, USA). Purified cDNA was ligated into PMD18-T Vector (Takara, Biotech, Japan) and sequenced completely from both directions with the equivalent specific primers (Table S1) designed based on their partial cDNA sequences. The open reading frames (ORF) of *CPG316, CPG860*, and *CPG4855* were amplified from *S. exigua* with the corresponding PCR primers (Table S1), respectively. The ORF PCR conditions for *CPG316, CPG860*, and *CPG 4855* genes were 3 min at 95 °C, followed by 40 cycles of 30 sec denaturation at 94 °C, 30 sec annealing at 59 °C, 60 °C and 58 °C, respectively, and 1 min extension at 72 °C and a final 10 min extension at 72 °C. The flanking PCR conditions follow the same procedure as ORF PCR. The full ORFs were cloned into pMD18-T Vector (Takara, Biotech, Japan) and at least three clones were sequenced for each fragment^38^.

### Sequence alignment and phylogenetic analysis

The amino acid sequences were blasted in NCBI database (https://www.ncbi.nlm.nih.gov/) using the blast-p option. The similar amino acid sequences were retrieved to use for construction of the phylogenetic tree with MEGA 7.0 and the same sequence aligned using ClustalX 2.1 with default settings and visualized in GeneDoc to show the conserved amino acids^39^. The percent similarity and identity were determined using (http://danio.mgh.harvard.edu/blast/wblast2.cgi?0) among the three cuticle protein genes *CPG316, CPG860*, and *CPG4855* and those of other insect species.

### Real-time PCR

Total RNA was isolated from the dissected integuments at six-time points during the intermolt period (endocuticle being synthesized) (3^rd^ instar, 4^th^ instar, 5^th^ instar, 0 h pupa, 2 h pupa, 24 h pupa) and reverse transcribed into cDNA as described above. The cDNAs were used as the templates for quantitative RT-PCR (qRT-PCR) analysis of *CPG316, CPG860*, and *CPG4855* expression using SYBR® Premix Ex TaqTM II kit (Takara, Biotech, Japan). Real-time PCR of *CPG316*, *CPG860*, *CPG4855*, *β-actin*, and *GAPDH* (*β-actin* and *GAPDH* were internal reference genes) were performed individually in a 20 μL reaction containing 2× SYBR® Premix Ex TaqTM II 10 μL, 10 *μ*M forward primer and reverse primer (0.8 μL each), 1 μL template cDNA, and 7.4 μL nuclease-free water. Specific primers (Table S1) were designed for real-time PCR of *CPG316, CPG860, CPG4855, β-actin* and *GAPDH* to generate the corresponding amplicons of 102 bp, 144 bp, 116 bp, 107 bp, and 174 bp, respectively. All real-time PCR reactions were performed in VIOX/SCIENTIFIC 96-well PCR plates. qTOWER 2.0 & 2.2 by Analytik Jena real-time PCR detection system was used as the fluorescence detector with the following common PCR conditions for the three genes: an initial denaturing cycle of 95 °C for 30 sec, followed by 40 cycles of denaturation at 95 °C for 5 sec, annealing at 60 °C for 10 sec and extension at 72 °C for 30 sec, and data collection and real-time analysis enabled at 72 °C. Melting curve analysis from 65 °C to 95 °C was run for each target and reference gene to ensure the absence of junk products. For the time points of 3^rd^ instar, 4^th^ instar, 5^th^ instar, 0 h pupa, 2 h pupa, 24 h pupa, there were three biological replicates of three integuments each for each gene, and each biological replicate was qRT-PCR-analyzed for three times. The expression levels of these three genes (*CPG316, CPG860, CPG4855*) at each time point were calculated and normalized to the geometric mean of the expression of the two reference genes (*β-actin* and *GAPDH*) with the 2−ΔCt method, where ΔCt = Ct target gene− Ct reference gene, and Ct refers to the cycle threshold of the gene^30,32^.

### dsRNA synthesis

According to the cloned ORF sequences of *CPG316, CPG860*, and *CPG4855* genes, we designed two pairs of gene-specific primers sense and antisense. The plasmid was extracted as a template, and then used the universal T7 promoter primer the sequence of amplified template for the synthesis of dsRNA with T7 RiboMAX™ Express RNAi System kit (Promega, USA). The same procedure was followed for dsRED as control, Sense primer pGEMTeasy with a T7 promoter sequence and antisense primer pGEMTeasy, follow by next pair of primers sense primer pGEMTeasy and antisense primer pGEMTeasy with the T7 promoter sequence. The dsRed reference template provided by the University of Arizona laboratory of Dr. Xianchun Li. The dsRNA were then purified with MEGA clear TM Kit (Ambion, USA).

### dsRNA injection

The 5^th^ instar beet armyworm larvae were starved for 24 h, then injected in the 2^nd^ to last dorsal abdominal segment. Two µl dsRNA of three different concentrations (100 ng/µl and 200 ng/µl, 500 ng/µl) for each cuticle gene, 500 ng/µl dsRed (control), were injected. Thirty insects were treated with each concentration; each treatment set was repeated 3 times. Phenotypes were observed after 96 h and the mortality of the treated insects was recorded daily.

After 96 h of treatment, three abnormal insects were selected for each gene and controls. The phenotypes of these samples were imaged with a digital camera after seven days with a high-resolution microscope (OLYMPUS SZX16).

### Statistical analysis

Data are presented as mean ± SD. One-way ANOVA with Duncan test was performed to test the significance of differences. Values sharing the different letters are significantly different at *P* < 0.05 (Duncan test). All the statistical tests were performed by SPSS (16.0).

## Results

### Sequence characterization

**Figure 1:**
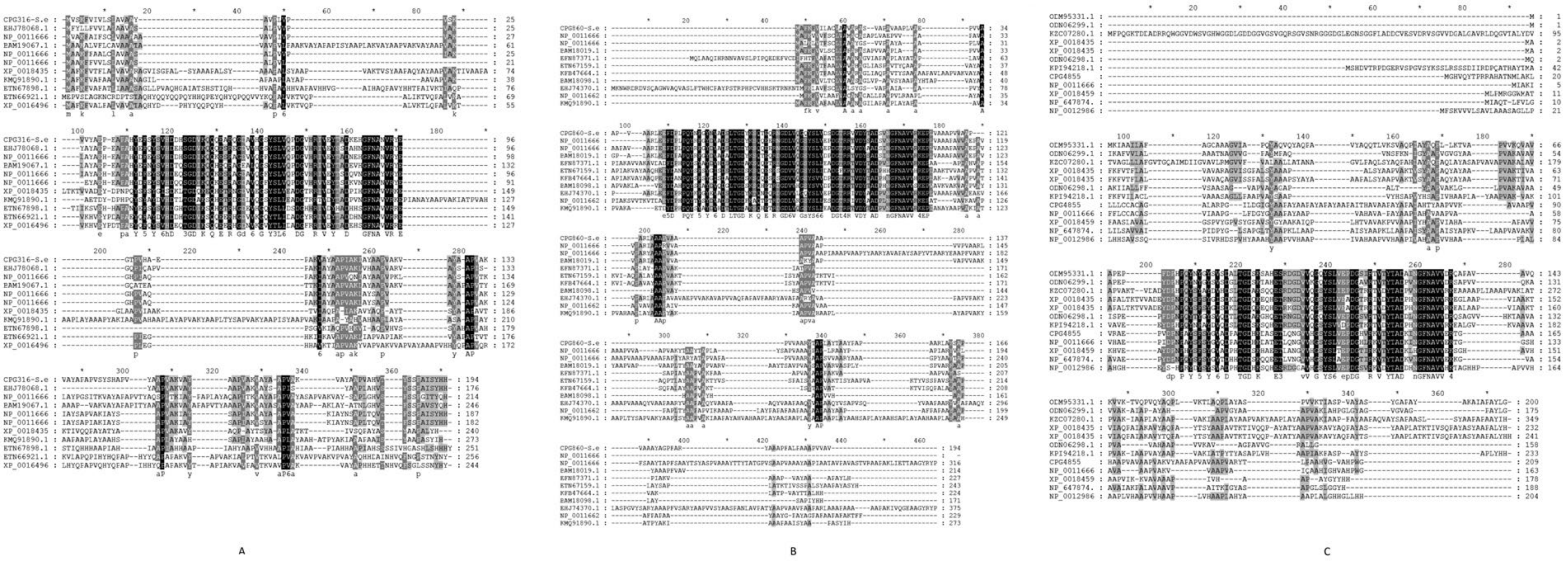
(**A**) Multiple alignments of the conserved region of the deduced amino acid sequence of *CPG316* with those of 331 other insect species. Black Shaded 100% conserved sequences are shown in the figure are active binding sites. (**B**) Multiple alignments of a conserved region of the deduced amino acid sequence of *CPG860* with those of 331 other insect species. Black Shaded 100% conserved sequences are shown in the figure are active binding sites. (**C**) Multiple alignments of the conserved region of the deduced amino acid sequence of *CPG4855* with those of 331 other insect species. Black Shaded 100% conserved sequences are shown in the figure are active binding sites.

The full-length cDNA sequence of *CPG316* was deposited in the gene bank, which was assigned with a KY554477 accession number. The *S. exigua CPG316* gene (722 bp) contained a 43 bp 5′UTR (untranslated region), an open reading frame (ORF) of 585 bp encoding 194 amino acids, and a 94 bp 3′UTR (Fig S1A). The corresponding amino acid sequence was searched against BLAST (https://www.ncbi.nlm.nih.gov/) use blast-p. Multiple alignments of *CPG316* conducted by Gendoc. The results revealed that it has the following identities and similarities with other cuticular proteins as follows: 69% identity and 84% similarity with *Bombyx mori* (NP_001166684.1), 68% identity and 83% similarity with *B. mori* (NP_001166633.1), 69% identity and 82% similarity with *Danaus plexippus* (EHJ78068.1), 64% identity and 75% similarity with *B. mori* (NP_001166683.1), 63% identity and 73% similarity with *Papilio polytes* (BAM19067.1), 45% identity and 55% similarity with *Anopheles darlingi* (ETN66921.1), 48% identity and 63% similarity with *A. darlingi* (ETN67898.1), 45% identity and 55% similarity with *Aedes aegypti* (XP_001649697.1), 44% identity and 54% similarity with *Culex quinquefasciatus* (XP_0018435071), and 41% identity and 52% similarity with *Lasius niger* (KMQ91890.1) (Fig. 1A).

The full-length cDNA sequence of *S. exigua CPG860* was translated and analyzed as described above for *CPG316*. The *CPG860* having accession code (KY554478), cDNA sequence (710 bp in total) contains a 49 bp 5′UTR, an open reading frame of 585 bp encoding 194 amino acids, and a 76 bp 3′UTR (Fig. S1B). From alignment of a region of conserved amino acids, *CPG860 S. exigua* have 68% identity and 73% similarity with *B. mori* (NP_001166689.1), 68% identity and 71% similarity with *Bombyx mori* (NP_001166690.1), 66% identity and 73% similarity with *P. xuthus* (BAM18019.1), 60% identity and 65% similarity with *D. plexippus* (EHJ74370.1), 56% identity and 65% similarity with *Harpegnathos saltator* (EFN87371.1), 55% identity and 64% similarity with *P. xuthus* (BAM18098.1), 53% identity and 61% similarity with *Nasonia vitripennis* (NP_001166273.1), 52% identity and 62% similarity with *L. niger* (KMQ91890.1), 50% identity and 50% similarity with *Anopheles sinensis* (KFB47664.1), 49% identity and 60% similarity with *A. darling* (ETN67159.1) (Fig. 1B).

The same procedure was followed for *S. exigua CPG4855*. The *CPG4855*, cDNA (accession code KY554479) and (868 bp in total) contains a 43 bp 5′UTR, consist of 630 bp ORF encoding 209 amino acids, and a 192 bp 3′UTR (Fig. S1C). Protein alignment analysis of *CPG4855* as done for the other two genes (Fig. 1C) shows that this gene has 68% identity and 74% similarity with *Papilio machaon* (KPJ08284.1), 67% identity and 72% similarity with *B. mori* (NP_001166686.1), 65% identity and 76% similarity with *Heliconius melpomene* (CBH09302.1), 65% identity and 74% similarity with *D. plexippus* (EHJ70419.1), 59% identity and 65% similarity with *Drosophila melanogaster* (NP_647874.1), 56% identity and 63% similarity with *C. quinquefasciatus* (XP_001845930.1), 52% identity and 60% similarity with *P. polytes* (NP_001298679.1), and 50% identity and 55% similarity with *Orchesella cincta* (NP_647874.1).

### Phylogenetic analysis

**Figure 2:**
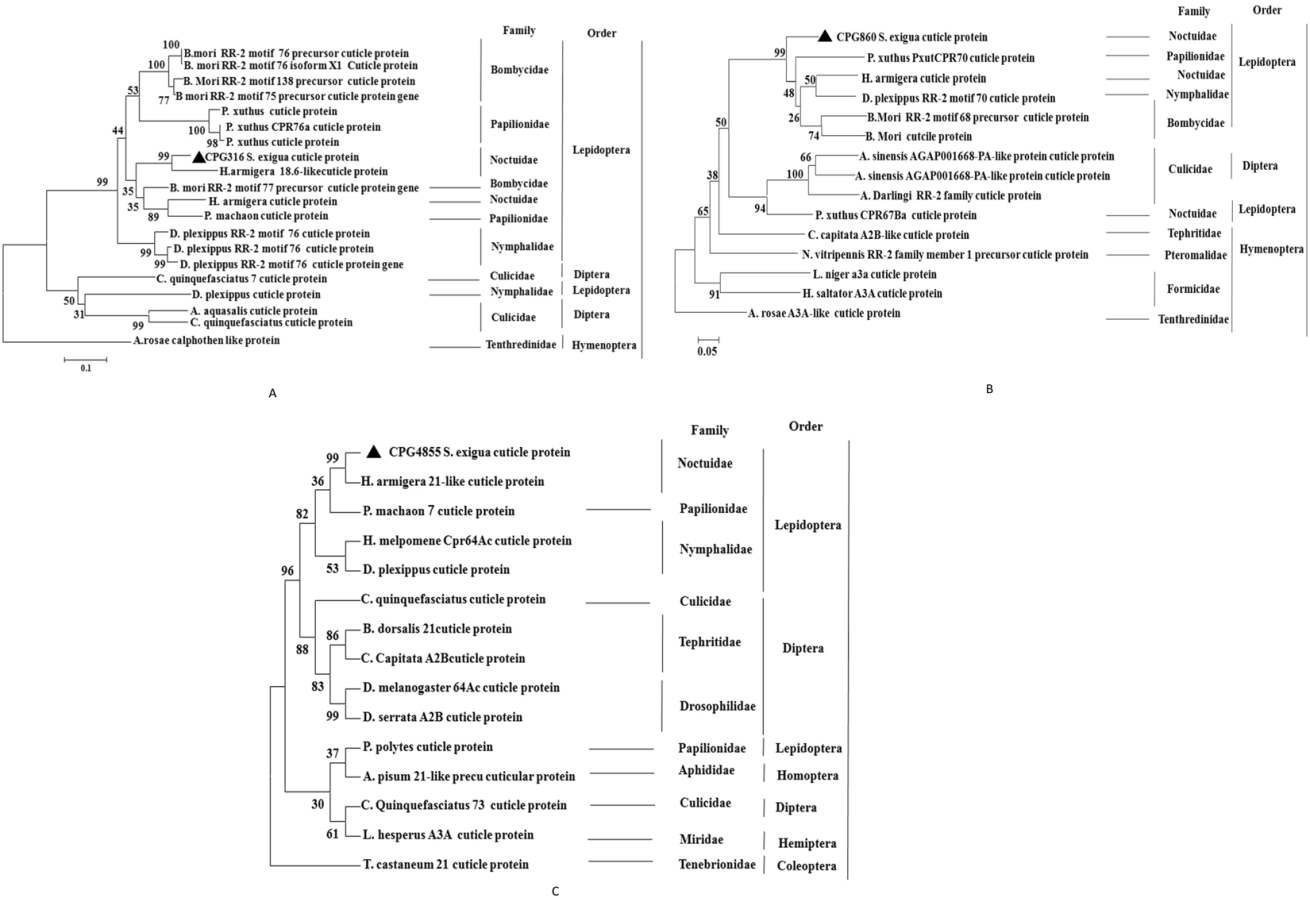
(**A**) Phylogenetic tree of insect *CPG316* based on its amino acid sequence. The phylogenetic tree was constructed using the neighbor-joining method with 1000 bootstrap replicates. The numbers at each tree node are the bootstrap values. (**B**) Phylogenetic tree of insect *CPG860* based on its amino acid sequence. The phylogenetic tree was constructed using the neighbor-joining method with 1000 bootstrap replicates. The numbers at each tree node are the bootstrap values. (**C**) Phylogenetic tree of insect *CPG4855* based on its amino acid sequence. The phylogenetic tree was constructed using the neighbor-joining method with 1000 bootstrap replicates. The numbers at each tree node are the bootstrap values.

The sequence of the conserved domain of *CPG316* is found in the same clade with Helicoverpa armigera, *B. mori* and *P. machaon* cuticle protein (Fig. 2A). The conserved region encoded by *CPG860* was found in the same clade with *P. xuthus, H. armigera, D. plexipus*, and *B. mori* cuticle proteins (Fig. 2B). The *CPG4855* was placed in the same clade with *H. armigera*, but little closed to *P. machaon* (Fig. 2C).

### Developmental expression

**Figure 3:**
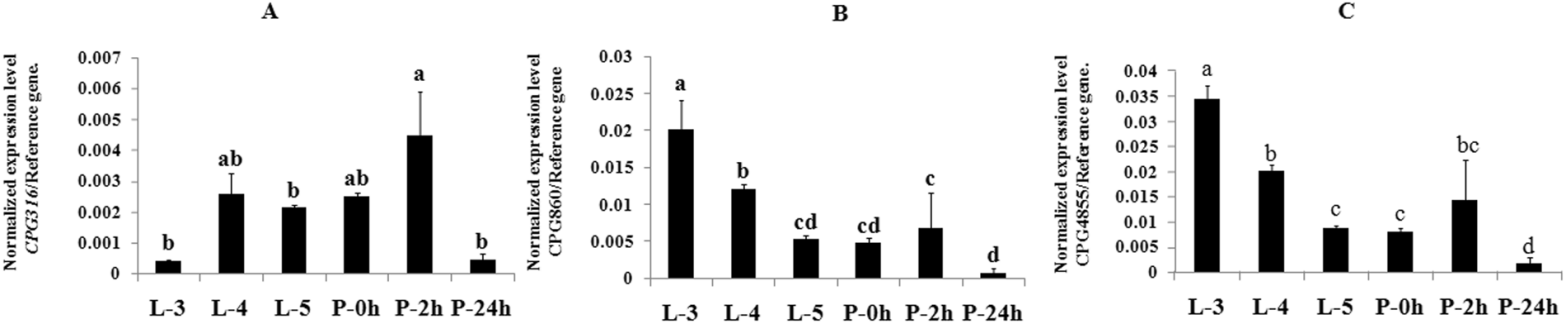
(**A**) Developmental expression of *CPG316* of *S.exigua*. The data and error bars represent the means and standard deviations of three biological replicates of three technical repeats each. Values sharing the same letter are not significantly different at *P* < 0.05 (Duncan test). (**B**) Developmental expression of *CPG860* of *S.exigua*. The data and error bars represent the means and standard deviations of three biological replicates of three technical repeats each. Values sharing the same letter are not significantly different at *P* < 0.05 (Duncan test). (**C**) Developmental expression of *CPG4855* of *S. exigua*. The data and error bars represent the means and standard deviations of three biological replicates of three technical repeats each. Values sharing the same letter are not significantly different at *P* < 0.05 (Duncan test).

*CPG316, CPG860*, and *CPG4855* transcripts were quantified in the qTOWER 2.0 & 2.2 by Analytik Jena real-time PCR detection system (Applied Biosystems). *Actin* and *GAPDH* genes were used as internal reference genes. All three genes were expressed at six-time points: 3^rd^, 4^th^, and 5^th^ instar larvae (L3, L4, and L5) and 0 h, 2 h, 24 h pupae (P 0 h, P 2 h, and P 24 h) (Fig. 3A,B,C). The results revealed significant differences in the normalized *CPG316* expression among the six developmental time points (Fig. 3A). *CPG316* is expressed in the 4^th^ instar larvae through the 0 h pupae at nearly constant levels, then increases in the 2 h pupae, only to fall again to a low level in the 24 h pupa that is similar to that in the 3^rd^ instar larvae. *CPG860* is expressed in all stages at 10-fold higher normalized levels than is *CPG316*. The highest level is observed in the 3^rd^ and 4^th^ instar larvae, intermediate level in the 5^th^ instar larvae to 2 h pupae and very low level in 24 h pupae (Fig. 3B). The level of normalized expression of *CPG4855* (Fig. 3C) was similar to that of *CPG860*. It is also highly expressed in 3^rd^ instar larvae, then the expression declined to a relatively low level in the 5^th^ instar larvae and 0 h pupae with a transient rise 2 h later, followed a decline to a very low level in the 24 h pupa.

### RNAi qRT-PCR down-regulation

Analysis of gene expression after dsRNA injection into *S. exigua* early 5^th^ instar larvae showed significant suppression of target transcripts in the dsRNA-injected larvae compared to the control group three genes tested with three different concentrations (*CPG316, CPG860*, and *CPG4855*). *CPG316* were suppressed with all concentrations of the dsRNA which are 5%, 7.1%, and 8.6% for 100 ng/µl, 200 ng/µl, and 500 ng/µl, respectively (Fig. S2A). In contrast, all concentration of *CPG860* dsRNA was significantly suppressive which were 23.89%, 28.94% and highly suppressed at the highest concentration which is 31.0% (Fig. S2B). In the case of *CPG4855*, the three concentrations produced 18.2%, 26.17%, and 28.54% suppression. All concentrations are significantly different as compared to the control (Fig. S2C).

### Phenotypes of *S. exigua* after treatment with dsRNA

**Figure 4:**
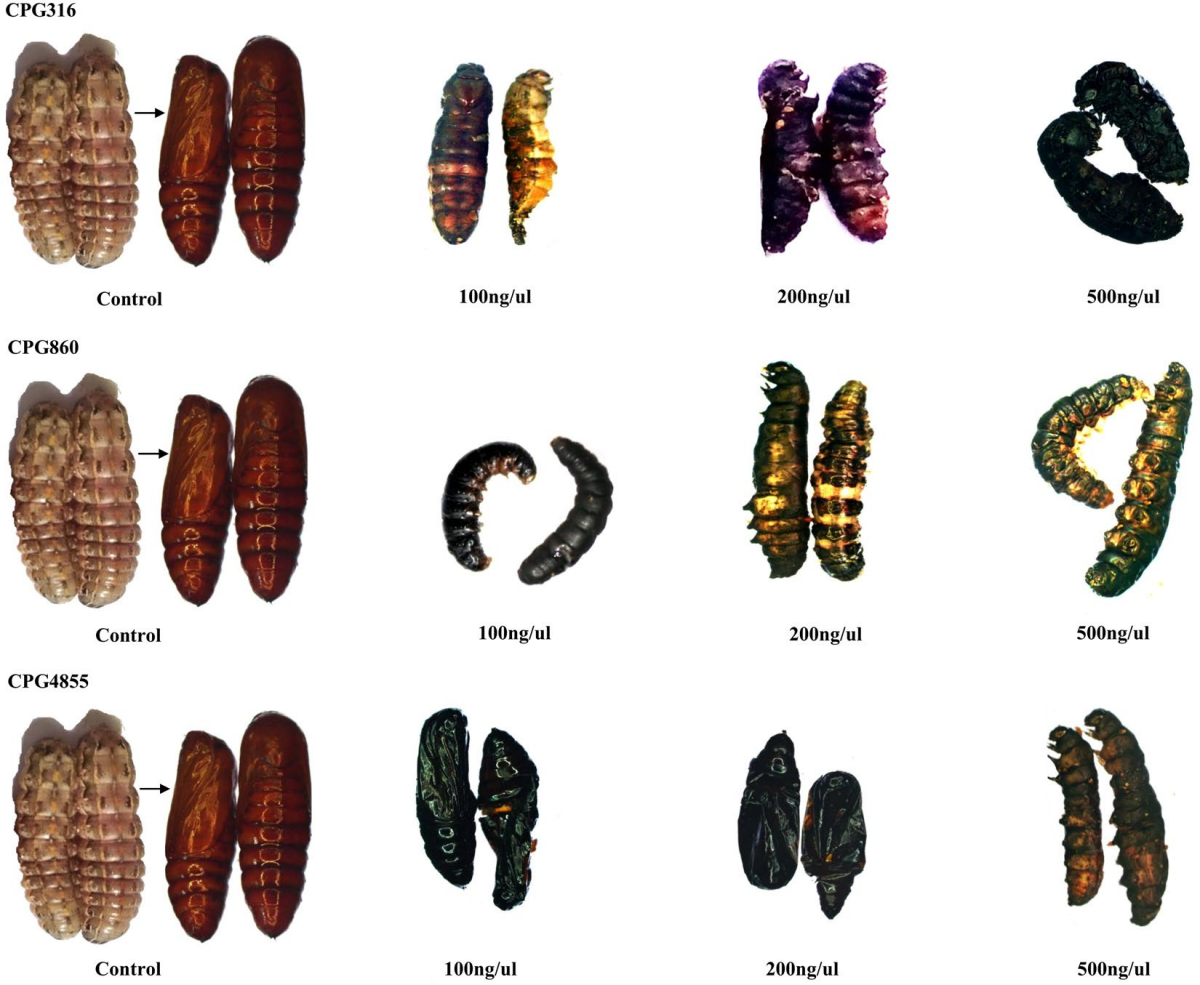
All genes phenotypes associated with three different concentrations of dsRNA as well (dsRed) as a control. (All three genes were separated with there names *CPG316*, *CPG860* and *CPG4855* while 100 ng/µl, 200 ng/µl, 500 ng/µl, indicate the concentrations of dsRNA).

Phenotypic changes were observed in treated larvae compared to controls seven days after the treatment. *CPG316* RNAi retarded growth and interfered with development. After injection of 100 ng/µl, the larvae developed to pupae but could not get ecdysis (Fig. 4). At the higher concentration of 200 ng/µl, some appeared to progress to the prepupal stage, but the larval cuticle became hard and black, and none ecdysis (Fig. 4) with 500 ng/µl RNAi, they all died in half larval and half pupae with hard black cuticle (Fig. 4) *CPG860* RNAi in both the 100 and 200 ng/µl concentrations caused black and hard cuticle on the tergites with some apparently forming prepupae, but do not get ecdysis (Fig. 4). With the highest concentration of dsRNA, the cuticle became completely black and hard and the larvae died (Fig. 4). After injection of 100 ng/µl and 200 ng/ul of *CPG4855* RNAi, larvae eclosed pupal stages but it becomes completely black and dead in the same stage (Fig. 4). At the higher doses, they died as half larvae and half pupal stage (Fig. 4).

### Percent mortality

While checking functional analysis through direct injection of dsRNA concentration wise 100, 200, and 500 ng/µl, respectively and dsRed 500 ng/µl as a control. Interference with all three cuticle genes caused high mortality as compared to control (Table S2).

## Discussion

In insects, there are various kinds of cuticle-related proteins, which differ in features and numbers in different species. These differences are due to numerous cuticular proteins and the sequence variation among them^12^. Cuticular proteins in a majority of insect species contain the Rebers and Riddiford consensus which is an extended form of a chitin binding region (R&R Consensus)^8,40,41^. Proteins with the Rebers and Riddiford consensus are divided into three classes, RR-1, RR-2, and RR-3 which have some association to the type of cuticle and its regions. There are more cuticular protein motifs reported in *Drosophila melanogaster* such as the Tweedle motif which was found in a protein that controls the larval body shape^9^. Chitin is a major biological polymer^42,43^ and its main role is a structural function in arthropod cuticles. Chitin interacts with cuticular protein to form an extremely regular structure. The chitin-binding domain in arthropod peritrophic membrane and chitinase proteins has been characterized and shown to bind chitin^43^.

In the current study, we have isolated three novel cuticular protein genes (*CPG316*, *CPG860*, and *CPG4855*) from *S. exigua* pupal integument. The alignment of the deduced amino acid sequences of our genes with those of other species^44^ shows identity and similarity in conserved domains. Phylogenetic analysis shows that they are most closely related to cuticular proteins that contain the RR-2 sequence^24,45^. This consensus sequence has been shown to have chitin-binding properties^40^.

Genes encoding cuticular proteins are thought to be good models to study the molecular mechanisms of signaling by ecdysteroids and juvenile hormones^46^. We have analyzed the expression pattern of all three isolated genes in different larval stages and at pupal time points. According to qRT-PCR results, *CPG316* mRNA was expressed in both 4^th^ and 5^th^ larval and pupal stages although at ten-fold lower levels than the other two genes. *CPG316* was highly expressed in the 2 h pupal stage and at very low levels in 3^rd^ instar larvae, whereas *CPG860* and *CPG4855* mRNAs were highly expressed in 3^rd^ instar larvae, then fell to low levels in 5^th^ instar larvae with a transient rise in the 2 h pupa. All three genes were expressed at very low levels in the 24 h pupa. Thus, it is likely that they are all contributing to the endocuticle of both larvae and pupae, although *CPG316* may play a somewhat different role from the other two.

In many organisms, to check the functional characterization of a gene, RNA interference (RNAi) has emerged as an influential tool. Use of RNAi shows major promise in biotechnology. Related applications consist of the capacity to avoid unnecessary transgene silencing in genetically engineered lines and the exploitation of various types of silencing to inactivate unwanted genes^47,48^. There are many techniques to introduce dsRNA but direct microinjection is the most general method for delivery of double-stranded RNA (dsRNA) into organisms. The knockdown ability of individual gene selectively through this reverse genetic technique has allowed many scientists to quickly expose the biological function of many genes inside many organisms by causing loss of specific function phenotypes^49^. RNA interference has proved its usefulness in functional genomic research on insects, but it also has considerable potential for the control of pest insects^33^.

Our studies using dsRNAs for the three isolated cuticle genes in final instar larvae showed that each suppressed the expression of the particular genes concentration wise significantly suppressed according to qRT-PCR. Yet all three different concentrations produced specific cuticular abnormalities which differed for the three genes. In all concentration of dsRNA, *CPG316* RNAi caused death in the pharate pupal stage. In contrast, *CPG860* RNAi caused the appearance of hard black cuticle on the tergites and also on the intersegmental membranes. These animals died before molting. *CPG4855* dsRNA caused complete cuticle blackness and hardening like pupae. Therefore, later on, these two genes apparently are involved in maintaining flexibility of the larval cuticle whereas *CPG316* may play a role in pupal cuticle formation that is needed for normal ecdysis.

In current research work, three new genes were cloned. All new genes have R&R consensus amino-acids and belong to the RR-2 consensus group. These genes are expressed in the larvae and early pupae but in different patterns. In contrast, Functional analysis results show that it effects on the cuticle in both larval and pupal stages, and necessary for cuticle development, flexibility, and metamorphosis. Further studies are necessary to determine whether these particular cuticular proteins have any role in tissues other than the integument.

## Electronic supplementary material

Supplementary data
