# Supplementary material for: Isolation and functional identification of three cuticle protein genes during metamorphosis of the beet armyworm, Spodoptera exigua
Source: Sci Rep. 2017 Nov 22;7:16061. doi: 10.1038/s41598-017-16435-w (PMC5700046; doi:10.1038/s41598-017-16435-w)
Supplement: Supplementary file 1 — Supplementary data [file 41598_2017_16435_MOESM1_ESM.doc]

**Isolation and functional identification of three cuticle protein genes during metamorphosis of the beet armyworm, *Spodoptera exigua***.

**Supporting information**

Max ORF starts at AA pos 111(may be DNA pos 331) for 113 AA(339 bases), MW=13602

10 20 30 40 50 60

1 ACAGTCAAGTATTGCACTTCAATACAGCAACACCAATCTAACAATGGTCTCCAAATTCGT

1 T V K Y C T S I Q Q H Q S N N G L Q I R

70 80 90 100 110 120

61 TATCGTTCTCTCCCTCGCCGTGGCGGCCTACGCCGTGCCCCTTGTTCCCGTCAGCAAGGT

21 Y R S L P R R G G L R R A P C S R Q Q G

130 140 150 160 170 180

121 GGTGTACGCCGAGCCTGAGGCGCCCGCTCACTACGAGTTCTCATACTCCGTGCACGATGA

41 G V R R A * G A R S L R V L I L R A R *

190 200 210 220 230 240

181 CCACAGCGGTGACGTGAAGCAGCAGCAGGAGGCGCGTCAAGGCGACGCCGTGCACGGCTC

61 P Q R * R E A A A G G A S R R R R A R L

250 260 270 280 290 300

241 GTACTCTCTCGTGCAGCCCGACGGTGTGCACCGCATCGTCGAGTACACCGCCGACAAGGA

81 V L S R A A R R C A P H R R V H R R Q G

310 320 330 340 350 360

301 GCACGGATTCAACGCCAACGTGCGCTATGAGGGCACCCCCGTCCACGCCGAGCCCGCCAA

101 A R I Q R Q R A L * G H P R P R R A R Q

370 380 390 400 410 420

361 GGTTGCCTACGCCGCCCCCATAGCCAAGATCGCCTACGCCGCTCCCGTTGCCAAGGTCGC

121 G C L R R P H S Q D R L R R S R C Q G R

430 440 450 460 470 480

421 CTACGCTGCTCCCGTTGCCAAAGTAGCCTACGCTCCAGCCCCAGTCTCTTACTCCCACGC

141 L R C S R C Q S S L R S S P S L L L P R

490 500 510 520 530 540

481 TCCAGTCTACGCTGCCCCCGTCGCCAAGGTCGCCTACGCCGCCCCCGTCGCCAAGGTCGC

161 S S L R C P R R Q G R L R R P R R Q G R

550 560 570 580 590 600

541 CTACGCCGCCCCCGTCGCCAAGGTCGCCTACGCTGCCCCCGTAGCGCACGTCACATACTC

181 L R R P R R Q G R L R C P R S A R H I L

610 620 630 640 650 660

601 CTCTCCTGCTATCTCCTACCACCACTAGACCAACATATTGTTAAACTTGTTGAAATGAAT

201 L S C Y L L P P L D Q H I V K L V E M N

670 680 690 700 710 720

661 AGGTATTATTAAATTATTATTTATTGACCTAAAAAAAAAAAAAAAAAAAAAAAAAAAAAA

221 R Y Y * I I I Y * P K K K K K K K K K K

721 AA

**Fig S1A**, Complete cDNA nucleotide sequence of *CPG316*

Max ORF starts at AA pos 120(may be DNA pos 358) for 100 AA(300 bases), MW=10975

10 20 30 40 50 60

1 ACATGGGGATAGTCGAACAAGACATCACAACTCCTCCTCACCAACAAAAATGGCCTTTAA

1 T W G * S N K T S Q L L L T N K N G L *

70 80 90 100 110 120

61 GTTCGTTATCCTCGCATGCGTGGCGGCTGTGGCCCGCGCCAGTGTGGCTCCAGCAGTGGC

21 V R Y P R M R G G C G P R Q C G S S S G

130 140 150 160 170 180

121 TGCTCCCCTCGTCGCGGAGCCCGTCGTAGCTGCACCTGTTGCAGCCAGGCTGGAGGAGTT

41 C S P R R G A R R S C T C C S Q A G G V

190 200 210 220 230 240

181 CGACCCGCTCCCACAGTACAACTTCGGATACAACGTGGCTGACTCGCTCACCGGCGACTA

61 R P A P T V Q L R I Q R G * L A H R R L

250 260 270 280 290 300

241 TAAAAGCCAAACTGAGCAGCGTAACGGAGACCTGGTTCAGGGTCAATACTCGTTGGTGGA

81 * K P N * A A * R R P G S G S I L V G G

310 320 330 340 350 360

301 CTCTGACGGCACTCGCCGTGTGGTCGACTACACTGCTGACTCAGTGAACGGTTTTAACGC

101 L * R H S P C G R L H C * L S E R F * R

370 380 390 400 410 420

361 AGTGGTGCGCAAAGAACCTTTGGTAGCTGCAGCTCCCGTGGTGGCTGCGCCAGCTCGTCT

121 S G A Q R T F G S C S S R G G C A S S S

430 440 450 460 470 480

421 CGCAGCCGCTCCCGTCGCTGCTGCTCCAGTGGCCGCGGCCCCCGTCGTCGCTGCGCGATA

141 R S R S R R C C S S G R G P R R R C A I

490 500 510 520 530 540

481 CACCGCTCCCCTCGCTTACACCGCGGCGTACCCCGCCCCAGCAGCTCGGCTGGCGTACAG

161 H R S P R L H R G V P R P S S S A G V Q

550 560 570 580 590 600

541 CGCGCCAGTAGCCGCCGCCTACGCCGGCCCATTCGCCCGGTATGCCGCCGCACCGTTCGC

181 R A S S R R L R R P I R P V C R R T V R

610 620 630 640 650 660

601 GTTGCCTGCTGCTGCACCCGTTGTAGCGGTCTAGACATTTATTATCCGGTTCCTGCATGA

201 V A C C C T R C S G L D I Y Y P V P A *

670 680 690 700 710

661 CTGACAACAATGGCAAATAAATTTTATACAACATCAAAAAAAAAAAAAAA

221 L T T M A N K F Y T T S K K K K

**Fig S1B**, Complete cDNA nucleotide sequence of *CPG860*

Max ORF starts at AA pos 175(may be DNA pos 523) for 66 AA(198 bases), MW=7137

10 20 30 40 50 60

1 CTATACGACTCACTATAGGGCAAGCAGTGGTATCAACGCAGAGTACATGGGACACGTGCA

1 L Y D S L * G K Q W Y Q R R V H G T R A

70 80 90 100 110 120

61 ATACACCCCACGACGTGCGCACGCCACTAACATGCTCGCTAAGTTGTTGTTGTTATGCTG

21 I H P T T C A R H * H A R * V V V V M L

130 140 150 160 170 180

121 TGCGTGTGCGGGCGCTCTGGCGCGGCCGGGGATACTGGACGGTATCGGCTACGCGGCTCC

41 C V C G R S G A A G D T G R Y R L R G S

190 200 210 220 230 240

181 AGCATATGCAGCGCCAGCTTATGCACCAGCTTACGCTGCGCCCGCGATTACTCACGCTGT

61 S I C S A S L C T S L R C A R D Y S R C

250 260 270 280 290 300

241 GGCTGCACCAGCGTACGCTCCCGCTATCGCACACACATACGCTGCACCCGTCGTTAAAGC

81 G C T S V R S R Y R T H I R C T R R * S

310 320 330 340 350 360

301 AGTTGCAGCTCCGGTCGTACGTGCCGAGCCCGTAGACCCTAATCCAGCTTACAGCTTCTC

101 S C S S G R T C R A R R P * S S L Q L L

370 380 390 400 410 420

361 ATACGGAGTAGCAGACCCCGCCACAGGTGACCACAAGGATGCTTCGGAGACGCTGCAGAA

121 I R S S R P R H R * P Q G C F G D A A E

430 440 450 460 470 480

421 TGGTGTGGTGCACGGATCTTACAGCCTAGTGGAACCTGACGGCCATGTCCGTAAGGTGAC

141 W C G A R I L Q P S G T * R P C P * G D

490 500 510 520 530 540

481 CTACACTGCTGACAAGATCAATGGATTCAACGCCGTGGTTGAAAGGACCGGAGCTGCTGC

161 L H C * Q D Q W I Q R R G * K D R S C C

550 560 570 580 590 600

541 CCACGCGGCACCAGTAGCAGTTGCCGCTCCAGTAGCCAAGGTTTACGCCGCGCCTGCGCC

181 P R G T S S S C R S S S Q G L R R A C A

610 620 630 640 650 660

601 TGTGGCGGTGGCTGCACCCGTCGCGAGGTACACCCTACCTGCTGCCCACGTTGGCGTTGC

201 C G G G C T R R E V H P T C C P R W R C

670 680 690 700 710 720

661 CCACCCGTGGGGTTAAACCCTACTACGACCACAAAGCGACCTAACATCTTGCTGAACGAC

221 P P V G L N P T T T T K R P N I L L N D

730 740 750 760 770 780

721 TAGAGGATTGTTTATGGATTAGATAGATGTCAACTCTAGATTGATGTAGAGAGTTTTCTA

241 * R I V Y G L D R C Q L * I D V E S F L

790 800 810 820 830 840

781 AAAAAACAAGGGTAATGATGAAATGTTTATTTAATTATTTTTGACTAAGATTGTTCGGTT

261 K K Q G * * * N V Y L I I F D * D C S V

850 860

841 CAGAATAGTTTGGTTAGAAATCATAGGT

281 Q N S L V R N H R

**Fig S1C**, Complete cDNA nucleotide sequence of *CPG4855.*

**
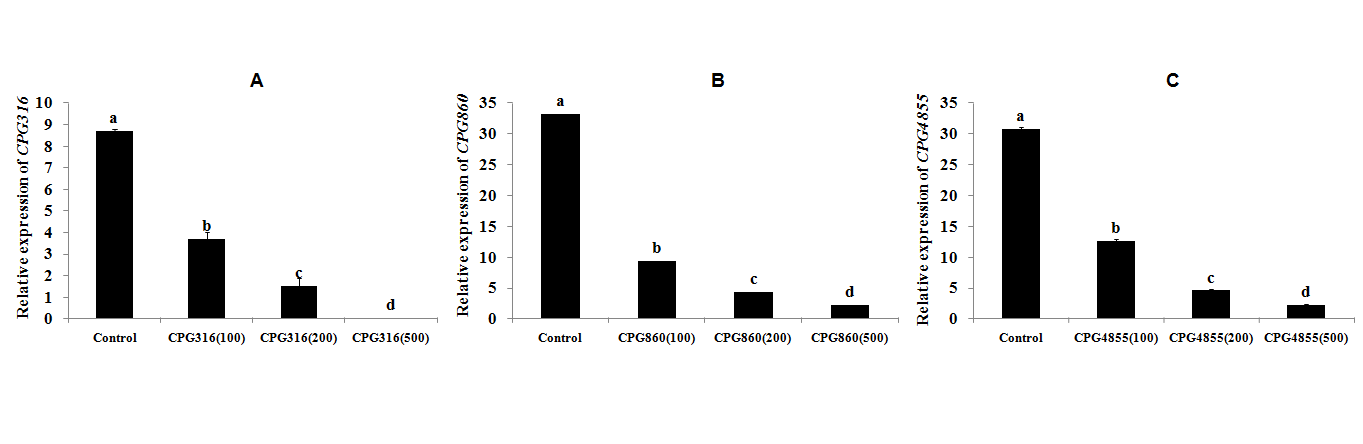
**

**Fig S2 :** Down-regulation of *Spodoptera exigua CPG316* RNA caused by injection of different concentrations of its dsRNA. The control was injected with dsRed.

**A:** Percent change level of *CPG316* at 100ng/ul, 200ng/ul, and 500ng/µl dsRNA concentrations is shown. The data and error bars represent the means and standard deviations of three biological replicates of three technical repeats each. Values sharing the different letter are significantly different at *P*<0.05 (Duncan test).

**B:** Percent change level of *CPG860* of *S.exigua.* Whereas *CPG860* shows gene name while 100ng/ul,200ng/ul, and 500ng/ul are dsRNA concentrations in the figure. The data and error bars represent the means and standard deviations of three biological replicates of three technical repeats each. Values sharing the different letter are significantly different at *P*<0.05 (Duncan test).

**C:** Percent change level of *CPG4855* of *S.exigua.*Whereas *CPG4855* shows gene name while 100ng/ul,200ng/ul, and 500ng/ul are dsRNA concentrations in the figure. The data and error bars represent the means and standard deviations of three biological replicates of three technical repeats each. Values sharing the different letter are significantly different at *P*<0.05 (Duncan test).

**Table S1,** All primers were used in the experiment.

(5′—3′)

| CPG316 -3GSP1 | CGTCAGCAAGGTGGTGTACG | |
| --- | --- | --- |
| CPG316 -3NGSP2 | GCCCGCTCACTACGAGTTCT | |
| CPG316 -5GSP1 | GAATCCGTGCTCCTTGTCG | |
| CPG316 -5NGSP2 | ACCGCTGTGGTCATCGTG | |
| CPG 316 -ORF Sense Primer | ATGGTCTCCAAATTCGTTAT | |
| CPG316 -ORF A.sense Primer | CTAGTGGTGGTAGGAGATAG | |
| CPG316- Sense Primer | CAAGGAGCACGGATTCAACG | |
| CPG316 – Sense Primer | TAGGCGATCTTGGCTATGGG | |
| ᵦ-actin-qRT Sense Primer | GGTATTGTGTTGGATTCCG | |
| ᵦ-actin- qRT A.Sense Primer | TGAGTAGCCCCTCTCTGTGAG | |
| CPG860 -3GSP1 | AAAGCCAAACTGAGCAGCGTA | |
| CPG860-3NGSP2 | CTGGTTCAGGGTCAATACTCGTT | |
| CPG860 -5GSP1 | TGCCGTCAGAGTCCACCAA | |
| CPG860 -5NGSP2 | GTTACGCTGCTCAGTTTGGC | |
| CPG 860 -ORF Sens Primer | ATGGCCTTTAAGTTCGTTATC | |
| CPG860 -ORF A.sense Primer | CTAGACCGCTACAACGGGTG | |
| CPG860- qRT Sense Primer | CAAGACATCACAACTCCTCCTC | |
| CPG860 – qRT A.Sense Primer | TTTGCCATTGTTGTCAGTCAT | |
| CPG4855 -5GSP1 | TCGCTTTGTGGTCGTAGTAGGGTTT | |
| CPG4855 -5NGSP2 | CCGTCAGGTTCCACTAGGCTGTAAGA | |
| CPG 4855 -ORF Sens Primer | ATGGGACACGTGCAATACACCC | |
| CPG4855 -ORF A.sense Primer | TTAACCCCACGGGTGGGCAACG | |
| CPG4855- qRT Sense Primer | GGTGTGGTGCACGGATCTT | |
| CPG5548 – qRT A.Sense Primer | GCAGCTCCGGTCCTTTCAAC | |
| Actine Sense Primer | ATCCTCCGTCTGGACTTGG | |
| Actine Anti sense | CGCACGATTTCCCTCTCA | |
| GAPDH sense.Primers | GACAACCACTCATCTATCTTCG | |
| GAPDH anti sense.Primer ` | AACATTTATCTCTACAACGCAATC | |
| CPG316 T7 promoter with sense primer | 5- GGATCCTAATACGACTCACTATAGGATGGTCTCCAAATTCGTTAT -3 | |
| CPG316 anti sense primer | 5- CTAGTGGTGGTAGGAGATAG -3 | |
| CPG316 Sense primers | ATGGTCTCCAAATTCGTTAT | |
| CPG316 T7 promoter anti sense primer | GGATCCTAATACGACTCACTATAGGCTAGTGGTGGTAGGAGATAG | |
| CPG860 T7 promoter with Sense primer | GGATCCTAATACGACTCACTATAGGATGGCCTTTAAGTTCGTTATC | |
| CPG860 anti Sense primer | | CTAGACCGCTACAACGGGTG |
| CPG860 T7 promoter with Sense primer | | ATGGCCTTTAAGTTCGTTATC |
| CPG860 T7 promoter with anti Sense primer | | ATACGACTCACTATAGGCTAGACCGCTACAACGGGTG |
| CPG4855 T7 promoter with sense primer | | ATCCTAATACGACTCACTATAGGATGGGACACGTGCAATACACCC |
| CPG4855 anti sense primer | | TTAACCCCACGGGTGGGCAACG |
| CPG4855 T7 promoter with sense primer | | GGATCCTAATACGACTCACTATAGGACACGTGCAATACACCC |
| CPG4855 anti sense primer | | TTAACCCCACGGGTGGGCAACG |
| T7 pGEM Teasy Sense Primer | | GGTGTAATACGACTCACTATAGG |
| pGEMTeasy A. Sense Primer | | CAAGCTATGCATCCAACGCGTTGGGAG |
| pGEMTeasy Sense Primer | | GGTGTAATACGACTCACTATAGGGCAAGCTATGCATCCAACGCGTTGGG |
| T7 pGEMTeasy A.Sense Primer | | CGAATTGGGCCCGGACGTCGCA |

**Table S2**. Percent mortality for the dsRNA experiments.

**
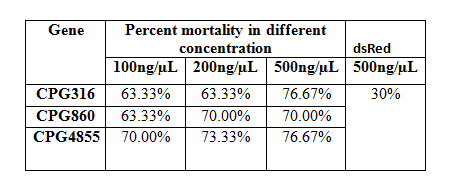
**
